# Automatic detection of contouring errors using convolutional neural networks

**DOI:** 10.1002/mp.13814

**Published:** 2019-09-26

**Authors:** Dong Joo Rhee, Carlos E. Cardenas, Hesham Elhalawani, Rachel McCarroll, Lifei Zhang, Jinzhong Yang, Adam S. Garden, Christine B. Peterson, Beth M. Beadle, Laurence E. Court

**Affiliations:** ^1^ The University of Texas Graduate School of Biomedical Sciences at Houston Houston TX 77030 USA; ^2^ Department of Radiation Physics Division of Radiation Oncology The University of Texas MD Anderson Cancer Center Houston TX 77030 USA; ^3^ Department of Radiation Oncology Division of Radiation Oncology The University of Texas MD Anderson Cancer Center Houston TX 77030 USA; ^4^ Department of Radiation Oncology The University of Maryland Medical System Baltimore MD 21201 USA; ^5^ Department of Biostatistics Division of Basic Sciences The University of Texas MD Anderson Cancer Center Houston TX 77030 USA; ^6^ Department of Radiation Oncology Stanford University School of Medicine Stanford CA 94305 USA

**Keywords:** autocontouring, contouring QA, convolutional neural network, deep learning, head and neck

## Abstract

**Purpose:**

To develop a head and neck normal structures autocontouring tool that could be used to automatically detect the errors in autocontours from a clinically validated autocontouring tool.

**Methods:**

An autocontouring tool based on convolutional neural networks (CNN) was developed for 16 normal structures of the head and neck and tested to identify the contour errors from a clinically validated multiatlas‐based autocontouring system (MACS). The computed tomography (CT) scans and clinical contours from 3495 patients were semiautomatically curated and used to train and validate the CNN‐based autocontouring tool. The final accuracy of the tool was evaluated by calculating the Sørensen–Dice similarity coefficients (DSC) and Hausdorff distances between the automatically generated contours and physician‐drawn contours on 174 internal and 24 external CT scans. Lastly, the CNN‐based tool was evaluated on 60 patients' CT scans to investigate the possibility to detect contouring failures. The contouring failures on these patients were classified as either minor or major errors. The criteria to detect contouring errors were determined by analyzing the DSC between the CNN‐ and MACS‐based contours under two independent scenarios: (a) contours with minor errors are clinically acceptable and (b) contours with minor errors are clinically unacceptable.

**Results:**

The average DSC and Hausdorff distance of our CNN‐based tool was 98.4%/1.23 cm for brain, 89.1%/0.42 cm for eyes, 86.8%/1.28 cm for mandible, 86.4%/0.88 cm for brainstem, 83.4%/0.71 cm for spinal cord, 82.7%/1.37 cm for parotids, 80.7%/1.08 cm for esophagus, 71.7%/0.39 cm for lenses, 68.6%/0.72 for optic nerves, 66.4%/0.46 cm for cochleas, and 40.7%/0.96 cm for optic chiasm. With the error detection tool, the proportions of the clinically unacceptable MACS contours that were correctly detected were 0.99/0.80 on average except for the optic chiasm, when contours with minor errors are clinically acceptable/unacceptable, respectively. The proportions of the clinically acceptable MACS contours that were correctly detected were 0.81/0.60 on average except for the optic chiasm, when contours with minor errors are clinically acceptable/unacceptable, respectively.

**Conclusion:**

Our CNN‐based autocontouring tool performed well on both the publically available and the internal datasets. Furthermore, our results show that CNN‐based algorithms are able to identify ill‐defined contours from a clinically validated and used multiatlas‐based autocontouring tool. Therefore, our CNN‐based tool can effectively perform automatic verification of MACS contours.

## 1 Introduction

Manual contouring is a time‐consuming process^1^, ^2^ and prone to inter‐ and even intrauser variabilities.^3^, ^4^, ^5^, ^6^, ^7^, ^8^ An autocontouring system can save the experienced user's time and reduce both inter‐ and intra‐user variabilities. However, an experienced user must review every contour generated from an autocontouring system before it would be used clinically. A previous study showed that automated contouring of head and neck structures can save 180 min per patient, but still requires 66 min to edit the automatically generated contours.^9^ Although the editing process takes significantly less time than the manual contouring process, this process still requires user's judgment, which can be biased and time‐intensive, and errors could still be missed. An automated contour review process that automatically flags suspicious cases could potentially be more objective, and provide additional time‐savings. Furthermore, the automatic review process would be an integral part of an automated radiation treatment planning system; we are currently developing such a system,^10^ which asks the users to review all the automatically generated contours every time a new treatment plan is created.

In this study, an autocontouring tool based on convolutional neural networks (CNN) was developed and tested. We then assess the error detection ability of our tool when applied to computed tomography (CT) scans with normal structures contoured using an in‐house atlas‐based contouring tool. This multi‐atlas based autocontouring system (MACS) is the primary contouring tool for a fully automated radiation treatment plan generator,^10^ and it has been used successfully for clinical^11^ and research purposes for several years.^12^, ^13^, ^14^, ^15^ Specifically, it is used to contour the normal structures for nearly all head and neck patients who receive radiotherapy at our institution, so the development of a CNN‐based autocontouring tool promises to augment and provide quality assurance to MACS.

Machine learning‐based contouring error detection algorithms have shown promising results in various radiation treatment sites. McIntosh et al.,^16^ used image features with conditional random forests algorithm to detect contouring errors in thoracic structures. Hui et al.,^17^ applied principal component analysis and Procrustes analysis on shapes of contours to detect contouring errors in male pelvis. Chen et al.,^18^ identified contouring errors in head and neck region by the geometric attribute distribution models. Furthermore, McCarroll et al.,^19^ developed a bagged tree classification model using contour features to predict the errors in MACS contours and achieved 0.63 accuracy. However, many of the erroneous MACS contours still have reasonably good shapes and relative position although the absolute positions are off by few millimeters to centimeters, and these errors are difficult to be detected by the machine learning‐based algorithms using the shapes and/or features of the contours. On the other hand, Beasley et al.,^20^ used volumetric overlap and distance between expert's contours and automatically generated contours to detect errors in the automatically generated contours, and was able to achieve AUC of 0.85–0.90 in detecting errors in parotids contours. As this approach is effective to detect even small offsets in contours, we implement the similar approach to detect errors in MACS contours by replacing the expert with the CNN‐based autocontouring tool.

The CNN algorithm was chosen to develop an autocontouring tool because other studies^21^, ^22^, ^23^, ^24^, ^25^, ^26^ have shown that CNN‐based models outperform most other machine learning‐based and model‐based algorithms in contouring head and neck structures. Zhu et al.,^22^ showed that their CNN‐based autocontouring algorithm for head and neck normal structures could achieve the equivalent performance with the best MICCAI 2015 challenge results with the atlas‐ and model‐based algorithms.^27^ Google DeepMind^23^ demonstrated that their CNN‐based autocontouring algorithm was able to achieve the near expert dosimetrist level accuracy and they also provided the ground truth contours on their test dataset to enable other autocontouring systems to benchmark the performance. However, most of the autocontouring tools were developed for internal use or commercial purpose and thus not publicly available. Therefore, we develop our own CNN‐based autocontouring tool and provide the performance of our tool with the benchmark data from Google DeepMind.

## 2 Materials and methods

The CNN‐based autocontouring tool was developed to generate contours for 16 head and neck normal structures: brain, brainstem, spinal cord, left and right cochlea, esophagus, left and right eyes, left and right lenses, mandible, optic chiasm, left and right optic nerves, and left and right parotids. These are the structures for which MACS can generate contours.

### 2.A. CNN‐based autocontouring model

#### 2.A.1. Training and validation data

The CT scans and the corresponding clinical contouring data of the 3495 patients who received external photon beam radiation treatment from September 2004 to June 2018 at the University of Texas MD Anderson Cancer Center were used as the training and validation data. Of these patients, 1169 had head and neck cancer, 1319 had brain cancer, and 1007 had thoracic or esophageal cancer. Contours for each structure were acquired independently to maximize the amount of data, and thus, the number of available structures in a single patient's data varied from 1 to 16. The total number of CT scans used for training and validation for each structure is given in Table 1. Of these scans, 80% were used for training, and 20% were used for cross‐validation. The data were collected solely on the basis of their structure labels, but manual review was conducted when two or more labels indicated the same structure in the same CT scan.

**Table 1:** The number of datasets used for training and validation for each structure.

Structure	Number of available clinical contours
Brain	1297
Brainstem	1702
Spinal cord	1332
Mandible	825
Esophagus	1134
Optic chiasm	983
Parotid (left, right)	997, 1016
Eyes (left, right)	978, 976
Lens (left, right)	1070, 1067
Optic nerve (left, right)	997, 996
Cochlea (left, right)	1664, 1662

**Figure 1:**
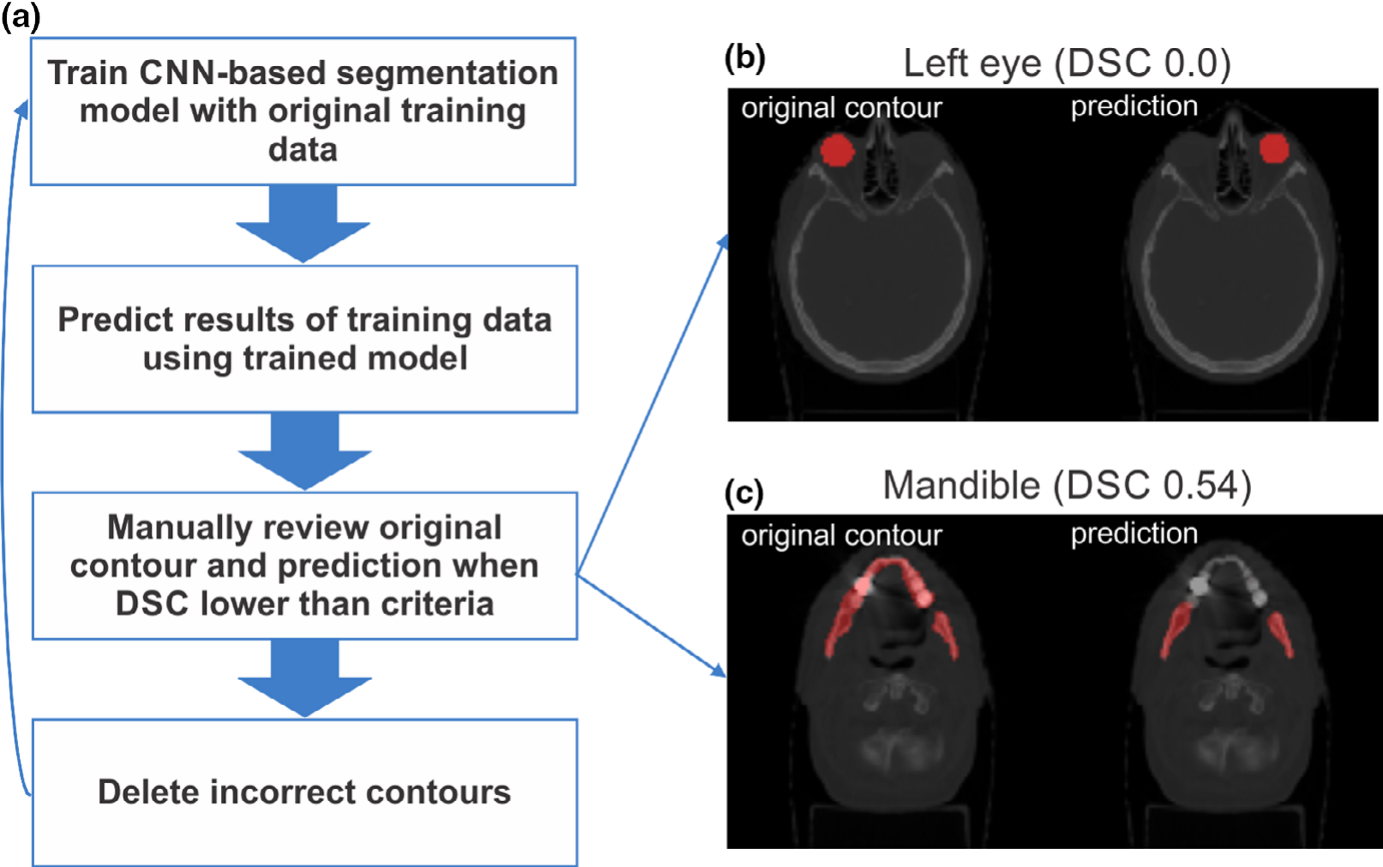
(a) The semiautomated method for data curation. When the Dice similarity coefficients was lower than required by the criteria, the original contour was reviewed manually. (b) Example of a structure labeled incorrectly (the right eye labeled as left eye). (c) Error in which the teeth were included as part of the mandible. [Color figure can be viewed at http://wileyonlinelibrary.com]

The data curation was performed semiautomatically as described in Fig. 1. First, the acquired clinical contours and the corresponding scans were given to a CNN‐based segmentation model for training. The model was trained until it could roughly segment the structures but was still underfitted. Then, the contours were automatically generated on the training data with the trained model, and the Sørensen–Dice similarity coefficients (DSCs)^14^ between the original training data and the predicted contours were calculated. When a calculated DSC was less than a certain value (0.6 for structures larger than an eye, 0.4 for smaller structures), the original contour was manually reviewed, and any of the incorrect clinical contours were removed from the training dataset. Once the entire set of training data was reviewed, we trained the model with the “refined” dataset again. This process was repeated two to three times, until all the significantly incorrect contours were eliminated from the training dataset.

Next, the training data were flipped and rotated for data augmentation. First, the structures were doubled by horizontal flipping. The paired structures had to be relabeled owing to their change in orientation from flipping (e.g., right eye becomes left eye after flipping). Then, the data were tripled by rotation around the longitudinal axis at two random angles between −30° and 30°.

#### 2.A.2. Training the CNN‐based segmentation model

**Figure 2:**
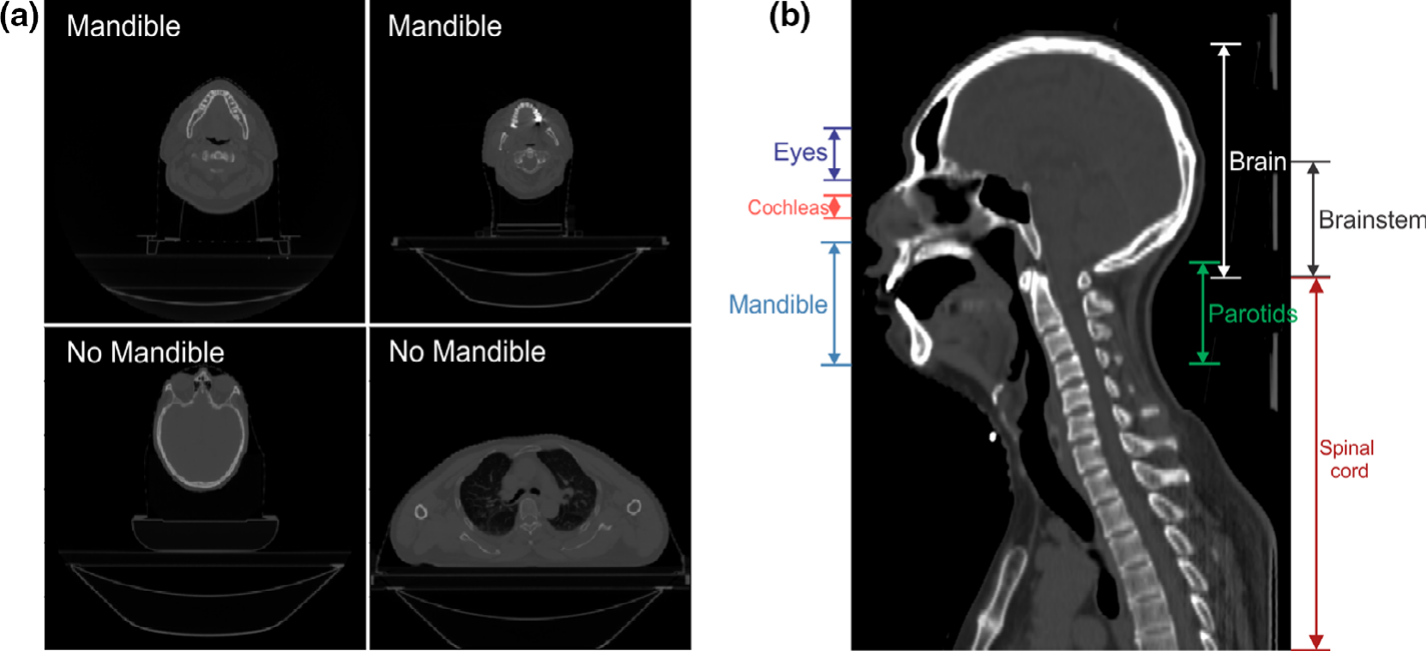
Application of the convolutional neural network‐based classification model to a computed tomography (CT) scan. The presence or absence of mandible on each CT slice was evaluated as shown in (a), and once the evaluation was done for every structure on all CT slices, the range of each structure in the longitudinal axis was selected for each patient as shown in (b). [Color figure can be viewed at http://wileyonlinelibrary.com]

The proposed model uses a combination of classification and segmentation CNN models. The Inception‐ResNet‐v2^28^ image classification model was trained to detect the existence of the structures in each CT slice. The classification results were used to determine the range of CT slices containing each structure, as shown in Fig. 2. The CT slices within the range, as shown in Fig. 2(b), were then given as an input to the segmentation models in the inference phase. The binary classifier was used to determine the presence or the absence of each structure in each image slice except for the brainstem and spinal cord. Instead, a three‐class classifier was used to select between the brainstem, spinal cord, and the absence of both structures, because they are physically connected in the axial direction. The ground truth for training and validation were created by labeling the slices containing clinical contour as presence.

We used two CNN‐based models for segmentation. The small structures (eyes, lenses, optic chiasm, optic nerves, and cochleas) were segmented using the V‐Net^29^ three‐dimensional (3D) CNN‐based model. The additional batch renormalization layers^30^ were applied to the end of every convolutional and up‐convolutional layer of the original V‐Net model. The other structures were segmented using the FCN‐8s,^31^ a two‐dimensional (2D) CNN‐based architecture. The batch renormalization layers were also added to the end of every layer in the FCN‐8s model.

The input size of 20 slices × 512 × 512 was used for the segmentation of the eyes and the associated structures (lenses, optic nerves, and optic chiasm). According to previous studies, the median (±standard deviation) diameter of a human eye is 24.9 ± 2.2 mm,^32^ and the mean heights of optic nerves and optic chiasm are 3.0 and 3.5 mm, respectively.^33^ Thus, we can assume that eyes and all associated structures are located within ±10 slices (±25 mm) from the longitudinal center of the eyes. An input image size of 20 slices × 512 × 512 was also chosen for cochlea segmentation for consistency, even though the cochleas were mostly covered within five CT slices. The rest of the structures were segmented with the FCN‐8s. The size of the input images was 512 × 512 × 1 channel. Only the CT slices that were classified to contain the structure of interest were transferred to the FCN‐8s model for segmentation. All of the classification and segmentation architectures were trained independently for each structure.

#### 2.A.3. Training parameters

The pixel sizes of the CT scans in the transverse plane varied from 0.53 to 1.37 mm, and the slice thicknesses varied from 1.0 to 3.75 mm, respectively. All data were resampled to have the same voxel size of 0.9766 mm × 0.9766 mm × 2.5 mm. The CT numbers lower than −1000 HU and higher than 3000 HU were clipped. Then, the CT numbers ranged from −1000 to 3000 HU were rescaled to the 0–255 pixel intensity range.

An NVIDIA DGX Station with four V100 GPUs was used to train our models. The loss function for the segmentation models was DSC as it was our metric to determine the accuracy of a segmentation model. A weighted cross‐entropy was used as a loss function for the classification model to compensate for the data imbalance between the number of slices with and without the organ of interest. The weight was determined to be the ratio the number of absence to the number of presence. The Adam optimizer^34^ was used as an optimization algorithm. The Adam optimizer's parameters beta1, beta2, and epsilon were set to 0.9, 0.999, and 10^−8^, respectively.

### 2.B. Evaluation

#### 2.B.1. Model accuracy

Twelve CT scans with baseline contouring atlas of the head and neck MACS and 162 CT scans from head and neck cancer patients who received proton radiation treatment at the University of Texas MD Anderson Cancer Center were used as the test data. Similar to the training and validation data, the number of available structures in a single patient's data varied from 1 to 16, and the total number of CT scans used for each structure is given in Table 2. The accuracy of the model was measured by the DSC and the Hausdorff distance^14^ between the model‐generated contours and the manual contours.

**Table 2:** The number of datasets used for testing for each structure.

Structure	Number of available clinical contours
Brain	108
Brainstem	126
Spinal cord	162
Mandible	117
Esophagus	111
Optic chiasm	48
Parotid (left, right)	168, 159
Eyes (left, right)	105, 106
Lens (left, right)	79, 80
Optic nerve (left, right)	67, 68
Cochlea (left, right)	77, 75

Also, 24 CT scans from The Cancer Imaging Archive (TCIA) with 14 normal structure contours (everything except esophagus and optic chiasm) were used as an external test dataset. The physician‐drawn contours for the TCIA dataset were provided by Google DeepMind.^23^ DeepMind also published the performance of their autocontouring model, which achieved near expert dosimetrist level performance on the TCIA dataset. We applied our CNN‐based model to the same TCIA data, calculated the DSC between our contours and the physician‐drawn contours from DeepMind, and compared the calculated DSC with the DeepMind's published DSC. We used a two‐tailed Student’s t‐test to detect any statistically significant difference between the two models, with significance defined by a *P* < 0.05.

#### 2.B.2. Automatic verification of automatically generated contours

We trained and tested our CNN‐based autocontouring system as an automatic verification tool with 48 CT scans with MACS contours and 12 CT scans with the baseline contouring atlas of the head and neck MACS, all of which were independent from the training dataset for the CNN‐based autocontouring system. The contours were scored by an experienced head and neck radiation oncologist on a scale of 1 to 3, where 1 was a clinically acceptable contour without editing (no error), 2 was a contour requiring minor editing (minor error), and 3 was a clinically unacceptable contour requiring major editing (major error),^35^ and the number of MACS contours for each score are given in Table 3. We then generated our CNN‐based model contours on these patients and calculated the DSC between the MACS contours and the CNN‐based model contours. Of these 60 patients, 40 patients were used for receiver operating characteristic (ROC) analysis based on their DSC and physician scores. We created two ROC curves per organ for two scenarios; (a) considering minor contouring errors to be clinically acceptable, so we would only detect major contouring errors, and (b) considering minor contouring errors to be clinically unacceptable, so we would detect both minor and major contouring errors. The DSC threshold, the minimum DSC to pass the automatic verification tool, was derived not to include any major errors for the scenario (a), and to include less than 30% of the minor errors for the scenario (b). Furthermore, area under the curves (AUCs) of the ROC curves were calculated to quantitate the relationship between DSC and physician scores.

**Table 3:** The number of multiatlas‐based autocontouring system (MACS) contours with each score for the 60 datasets.

Structure	No errors	Training dataset		NA*	No errors	Testing dataset		NA*
		Minor errors	Major errors			Minor errors	Major errors	
Brain	28	2	5	5	13	2	1	3
Brainstem	26	7	2	5	13	3	1	3
Cochleas	28	29	13	10	13	16	5	6
Lenses	43	7	30	0	26	2	12	0
Mandible	16	12	8	4	8	5	4	3
Optic nerves	28	25	17	10	15	8	11	6
Eyes	35	20	19	6	17	11	8	4
Parotids	39	18	13	10	21	9	4	6
Spinal cord	27	6	2	5	14	3	0	1
Esophagus	11	9	20	0	10	5	4	1
Optic chiasm	15	8	12	5	6	8	3	3

NA*: Only a subset of MACS contours available for some patients.

To test the DSC thresholds, we calculated the sensitivity and specificity on the other 20 patient data. We defined clinically acceptable contours to be positive and clinically unacceptable contours to be negative. Therefore, the sensitivity measures the proportion of the clinically acceptable contours that are correctly detected as such and the specificity measures the proportion of the clinically unacceptable contours that are correctly detected as such. As the DSC thresholds were derived independently for each scenario, the sensitivity and specificity were calculated independently for each scenario with corresponding thresholds as well.

## 3 Results

### 3.A. Model accuracy

The average DSCs and Hausdorff distances between our model contours and clinical contours are calculated in Table 4. For the large structures created with FCN‐8 architecture, the DSCs were higher than 80.7%, and for the small structures created with the V‐Net model, the DSCs were higher than 65.2% except for the optic chiasm.

**Table 4:** Sørensen–Dice similarity coefficients (in percentage) and Hausdorff distance (in cm) between our convolutional neural network‐based model and clinical contours from 174 test patients.

Structure	DSC	SD	Hausdorff Distance	SD
Brain	98.4	0.3	1.23	1.52
Brainstem	86.4	7.9	0.88	1.96
Cochlea, left	65.2	11.9	0.47	0.36
Cochlea, right	67.6	13.1	0.45	0.35
Lens, left	72.9	13.9	0.42	1.10
Lens, right	70.4	14.5	0.35	0.67
Mandible	86.8	3.3	1.28	0.95
Optic nerve, left	67.9	9.2	0.69	0.77
Optic nerve, right	69.3	8.5	0.74	0.72
Eye, left	88.8	3.7	0.49	1.42
Eye, right	89.3	3.6	0.35	0.10
Parotid, left	82.6	6.4	1.34	0.56
Parotid, right	82.7	4.8	1.39	0.58
Spinal cord	83.4	6.4	0.71	1.31
Esophagus	80.7	7.0	1.08	1.09
Optic chiasm	40.7	13.9	0.96	0.40

DSC, Dice similarity coefficient; SD: standard deviation.

The DSCs between our model contours and the DeepMind physician‐drawn contours and between the DeepMind model contours and the DeepMind physician‐drawn contours are in Table 5. The differences between the models for both lenses, both parotids, the left optic nerve, the left eye, and the spinal cord were not statistically significant, and our model performed better than DeepMind's in contouring the brainstem. Our model performed worse than DeepMind's in the brain, both cochleas, the mandible, the right optic nerve, and the right eye, but the differences were smaller than 3.5% except both cochleas. The differences in the standard deviations of the DSCs of the two models were small (<2%) except for the brainstem and lenses, where DeepMind's outcomes were more sparsely distributed than our model’s outcomes. The Hausdorff distance between our model contours and DeepMind physician‐drawn contours are in Table 6, and the average Hausdorff distances were <1.78 cm for all structures except for the brain.

**Table 5:** Sørensen–Dice similarity coefficients (in percentage) between our convolutional neural network‐based model and DeepMind's physician‐drawn contours on the TCIA data and between DeepMind's model and DeepMind's physician‐drawn contours.

Structure	Model	0522c0017	0522c0057	0522c0161	0522c0226	0522c0248	0522c0251	0522c0331	0522c0416	0522c0419	0522c0427	0522c0457	0522c0479	0522c0629	0522c0768	0522c0770	0522c0773	0522c0845	TCGA‐CV‐7236	TCGA‐CV‐7243	TCGA‐CV‐7245	TCGA‐CV‐A6JO	TCGA‐CV‐A6JY	TCGA‐CV‐A6K0	TCGA‐CV‐A6K1	Mean	SD	Diff (%)	*P* value
Brain	Ours	97.0	95.7	96.4	96.9	96.9	96.0	97.0	97.2	96.8	97.4	97.2	97.0	96.8	96.8	97.6	97.2	96.3	97.1	97.1	97.0	97.3	97.4	96.8	97.5	96.9	0.5	−2.1	0.000
	DM	98.9	98.8	99.2	98.7	98.8	98.2	98.8	99.2	98.8	98.8	99.1	98.9	99.1	99.2	98.8	98.8	99.0	99.3	99.3	98.9	99.2	99.2	99.0	99.0	99.0	0.2		
Brainstem	Ours	89.8	88.8	86.1	84.2	91.8	87.4	85.0	87.0	88.9	84.9	90.1	88.9	88.4	91.0	92.3	91.1	84.0	83.7	83.7	82.1	89.6	90.5	87.3	88.9	87.7	2.9	8.6	0.000
	DM	78.0	69.2	82.9	73.2	67.6	70.1	71.4	90.8	78.1	63.6	91.2	69.6	89.1	88.9	66.0	59.3	86.9	87.4	87.1	85.5	82.6	86.3	86.0	87.9	79.1	9.8		
Cochlea, left	Ours	38.5	33.5	41.4	43.7	33.6	53.3	31.3	43.5	23.9	37.3	28.6	46.8	47.0	30.3	38.3	30.7	34.4	43.4	43.4	40.2	48.9	38.1	52.0	46.3	39.5	7.7	−42.3	0.000
	DM	71.2	81.0	79.2	90.6	79.1	89.6	81.8	82.4	81.0	74.1	80.0	87.3	87.0	81.2	85.2	76.0	91.9	88.9	76.2	86.3	70.9	94.7	70.8	76.5	81.8	6.8		
Cochlea, right	Ours	51.9	28.4	39.3	34.9	34.7	59.6	38.6	43.0	28.7	38.5	43.7	35.0	56.8	30.8	40.2	39.1	45.0	36.7	36.7	46.6	59.6	40.2	52.4	51.7	42.2	9.1	−38.6	0.000
	DM	83.0	85.7	90.3	83.6	86.4	89.6	77.6	82.8	71.7	77.3	68.8	80.0	82.5	83.9	83.3	86.8	78.8	81.0	55.9	89.8	71.6	90.9	80.0	77.8	80.8	7.9		
Lens, left	Ours	91.1	84.2	84.2	71.4	85.3	83.3	78.4	78.1	79.8	77.2	78.7	54.1	88.6	82.2	79.3	85.4	69.7	87.2	87.2	86.2	85.5	76.9	82.5	90.1	81.1	7.9	1.1	0.793
	DM	85.5	77.7	73.8	87.3	87.0	91.4	79.6	60.0	82.6	86.2	83.5	0.0	91.3	86.0	65.0	90.9	79.4	91.3	82.6	87.9	88.1	87.5	86.2	89.4	80.0	18.8		
Lens, right	Ours	81.4	68.8	85.1	85.9	84.6	80.0	78.3	45.5	87.7	71.5	72.4	34.9	85.9	65.2	69.5	83.3	72.2	82.9	82.9	83.3	91.1	88.3	82.1	85.5	77.0	13.4	3.9	0.546
	DM	82.2	0.0	66.7	81.7	86.7	85.5	81.1	0.0	84.7	83.3	87.0	0.0	78.4	82.2	76.9	81.6	91.4	76.6	84.4	92.1	91.8	88.6	80.4	90.5	73.1	28.8		
Mandible	Ours	93.3	92.4	86.7	94.2	91.0	86.2	88.0	89.1	91.1	90.1	90.0	88.0	91.8	85.8	92.1	91.0	87.6	92.8	92.8	89.4	91.5	90.6	91.0	91.7	90.3	2.3	−3.5	0.000
	DM	92.2	94.0	93.8	91.3	95.0	95.4	91.1	92.3	95.4	94.4	96.4	92.4	93.8	91.0	93.2	91.9	94.8	94.6	92.3	95.5	96.5	93.3	95.4	95.4	93.8	1.7		
Optic nerve, left	Ours	78.7	76.4	78.3	77.6	73.4	66.5	72.2	72.5	66.8	73.4	81.6	75.7	75.4	76.2	75.8	71.1	76.7	75.4	75.4	85.6	82.4	80.2	81.1	64.5	75.5	5.1	−2.6	0.094
	DM	68.3	73.5	79.5	77.9	81.9	70.7	71.4	70.9	75.2	78.6	75.6	80.6	83.2	74.8	71.1	84.1	80.7	78.0	81.0	85.4	85.5	82.2	84.6	78.7	78.1	5.2		
Optic nerve, right	Ours	76.8	71.1	72.3	75.4	79.6	73.0	74.3	71.1	70.3	71.6	75.1	81.5	65.8	74.6	69.8	70.9	74.5	79.0	79.0	64.6	81.7	78.4	79.3	58.2	73.7	5.6	−3.3	0.036
	DM	70.4	75.3	76.6	78.6	77.8	75.2	72.8	73.1	84.3	79.6	81.4	77.7	71.7	76.7	79.2	81.8	78.0	80.6	77.4	73.0	77.4	82.4	85.9	61.4	77.0	5.1		
Eye, left	Ours	92.2	89.3	92.0	91.9	86.6	94.5	84.7	84.2	89.9	91.5	92.5	89.1	92.4	89.4	90.2	87.8	87.7	90.3	90.3	93.0	90.9	92.9	94.1	93.3	90.4	2.7	−1.1	0.139
	DM	90.1	92.1	91.1	91.2	92.7	89.4	92.3	90.5	92.6	93.8	94.3	93.4	94.5	88.1	91.7	84.4	93.2	92.1	90.4	90.6	92.6	91.4	90.9	93.1	91.5	2.2		
Eye, right	Ours	88.3	91.0	91.5	91.4	90.2	91.9	86.0	87.7	91.8	90.0	92.5	84.9	90.5	91.2	91.9	91.0	89.5	91.8	91.8	91.5	91.2	93.5	90.9	94.0	90.7	2.1	−1.4	0.015
	DM	92.7	93.6	91.1	93.6	94.4	90.4	92.1	90.1	94.2	94.5	92.9	93.5	93.4	90.0	92.2	86.3	94.1	91.4	93.0	91.0	90.0	91.1	93.4	92.2	92.1	1.9		
Parotid, left	Ours	74.3	82.9	84.5	85.8	82.7	86.2	77.6	83.3	69.8	88.0	79.7	79.9	86.2	84.8	81.8	87.4	84.0	84.4	84.4	86.2	78.3	72.7	86.1	88.2	82.5	4.9	−0.7	0.629
	DM	81.0	78.6	81.2	86.4	80.8	86.4	66.8	87.6	72.2	85.8	86.3	83.2	85.8	87.2	83.8	82.1	87.2	82.8	88.9	86.5	82.2	75.3	89.7	89.0	83.2	5.5		
Parotid, right	Ours	71.1	81.0	83.8	89.4	87.3	82.0	72.7	82.7	73.7	86.1	88.2	81.0	84.5	84.9	79.6	88.5	82.2	85.7	85.7	85.4	75.5	82.5	85.9	86.3	82.7	5.0	−1.3	0.320
	DM	84.4	80.2	83.6	88.6	87.0	79.6	82.1	86.4	72.6	86.4	91.4	84.2	85.4	86.7	82.9	85.9	82.6	84.2	84.6	84.4	77.5	84.7	86.2	85.1	84.0	3.8		
Spinal cord	Ours	76.4	80.4	66.2	86.5	85.8	80.8	84.3	82.2	56.6	76.7	85.9	76.3	84.6	82.0	83.9	87.6	86.2	66.8	66.8	71.3	78.2	84.3	84.0	83.4	79.1	8.1	−0.9	0.679
	DM	76.6	84.4	66.0	86.2	86.5	82.4	85.1	84.5	56.3	78.1	89.9	76.7	86.3	79.8	81.8	88.6	86.9	68.0	81.0	70.1	78.7	83.6	83.8	79.1	80.0	8.0		

Ours: our CNN‐based model; DM: DeepMind's model; SD: standard deviation; Diff: difference in DSC between the models.

**Table 6:** Hausdorff distance (in cm) between our convolutional neural network‐based model and DeepMind's physician‐drawn contours on the TCIA data.

Structure	0522c0017	0522c0057	0522c0161	0522c0226	0522c0248	0522c0251	0522c0331	0522c0416	0522c0419	0522c0427	0522c0457	0522c0479	0522c0629	0522c0768	0522c0770	0522c0773	0522c0845	TCGA‐CV‐7236	TCGA‐CV‐7243	TCGA‐CV‐7245	TCGA‐CV‐A6JO	TCGA‐CV‐A6JY	TCGA‐CV‐A6K0	TCGA‐CV‐A6K1	Mean	SD	Maximum
Brain	3.48	3.73	3.85	3.39	3.84	3.50	3.37	3.55	3.31	3.43	3.80	3.59	3.71	3.52	3.76	3.68	3.42	3.50	3.50	3.48	3.78	3.51	3.51	3.49	3.57	0.16	3.85
Brainstem	0.55	0.52	0.90	0.78	0.43	0.76	0.60	0.61	0.45	0.59	0.41	0.47	0.62	0.55	0.4	0.59	0.62	0.59	0.59	0.90	0.48	0.60	0.70	0.62	0.60	0.14	0.90
Cochlea, left	0.55	0.67	0.37	0.73	0.67	0.57	0.75	0.49	0.75	0.80	0.76	0.59	0.65	0.65	0.73	0.64	0.82	0.62	0.62	0.63	0.58	0.75	0.49	0.70	0.65	0.11	0.82
Cochlea, right	0.49	0.87	0.71	0.77	0.75	0.57	0.70	0.39	0.88	0.70	0.71	0.79	0.63	0.72	0.74	0.68	0.63	0.69	0.69	0.66	0.53	0.75	0.49	0.53	0.67	0.12	0.88
Lens, left	0.13	0.16	0.22	0.25	0.20	0.17	0.23	0.22	0.16	0.21	0.15	0.41	0.15	0.15	0.17	0.21	0.24	0.17	0.17	0.16	0.20	0.23	0.23	0.14	0.20	0.06	0.41
Lens, right	0.20	0.22	0.27	0.16	0.14	0.14	0.25	0.29	0.13	0.21	0.20	0.47	0.23	0.23	0.23	0.22	0.30	0.25	0.25	0.23	0.20	0.14	0.21	0.16	0.22	0.07	0.47
Mandible	1.06	1.63	1.65	1.61	1.11	1.76	1.05	1.87	1.40	1.41	1.56	1.16	1.56	0.7	0.76	0.66	0.86	1.39	1.39	1.80	1.14	1.90	1.66	1.15	1.34	0.37	1.90
Optic nerve, left	0.43	0.49	0.30	0.26	0.83	0.36	0.33	0.41	1.78	0.29	0.23	0.35	0.60	0.27	0.33	1.18	0.32	0.49	0.49	0.22	0.28	0.39	0.24	0.81	0.49	0.36	1.78
Optic nerve, right	0.43	0.83	0.65	0.35	0.24	0.41	0.63	0.83	0.64	0.37	0.24	0.24	0.77	0.41	0.48	0.31	0.59	0.40	0.40	0.35	0.26	0.37	0.31	0.60	0.46	0.19	0.83
Eye, left	0.27	0.27	0.23	0.27	0.50	0.24	0.61	0.52	0.26	0.43	0.25	0.43	0.25	0.29	0.29	0.31	0.49	0.27	0.27	0.25	0.32	0.23	0.27	0.31	0.33	0.11	0.61
Eye, right	0.32	0.46	0.29	0.27	0.37	0.27	0.45	0.48	0.27	0.54	0.32	0.50	0.30	0.29	0.33	0.24	0.5	0.29	0.29	0.32	0.31	0.23	0.29	0.29	0.34	0.09	0.54
Parotid, left	2.54	1.40	2.52	1.40	1.31	0.97	1.77	2.00	1.66	1.15	1.64	0.91	0.63	1.04	1.22	2.30	1.55	2.76	2.76	2.77	2.37	1.91	1.03	0.58	1.67	0.69	2.77
Parotid, right	2.73	1.55	2.15	1.16	0.75	2.31	2.10	2.02	2.19	1.84	1.42	2.82	0.88	1.32	1.47	0.99	3.23	1.00	1.00	1.22	2.98	2.23	2.00	1.24	1.78	0.71	3.23
Spinal cord	0.54	0.64	0.51	0.84	0.37	0.77	0.55	0.52	0.46	0.54	0.37	0.40	0.55	0.35	0.34	0.32	0.56	0.42	0.42	0.35	0.39	0.46	0.55	0.29	0.48	0.14	0.84

SD: standard deviation.

### 3.B. Automatic verification of automatically generated contours

**Figure 3:**
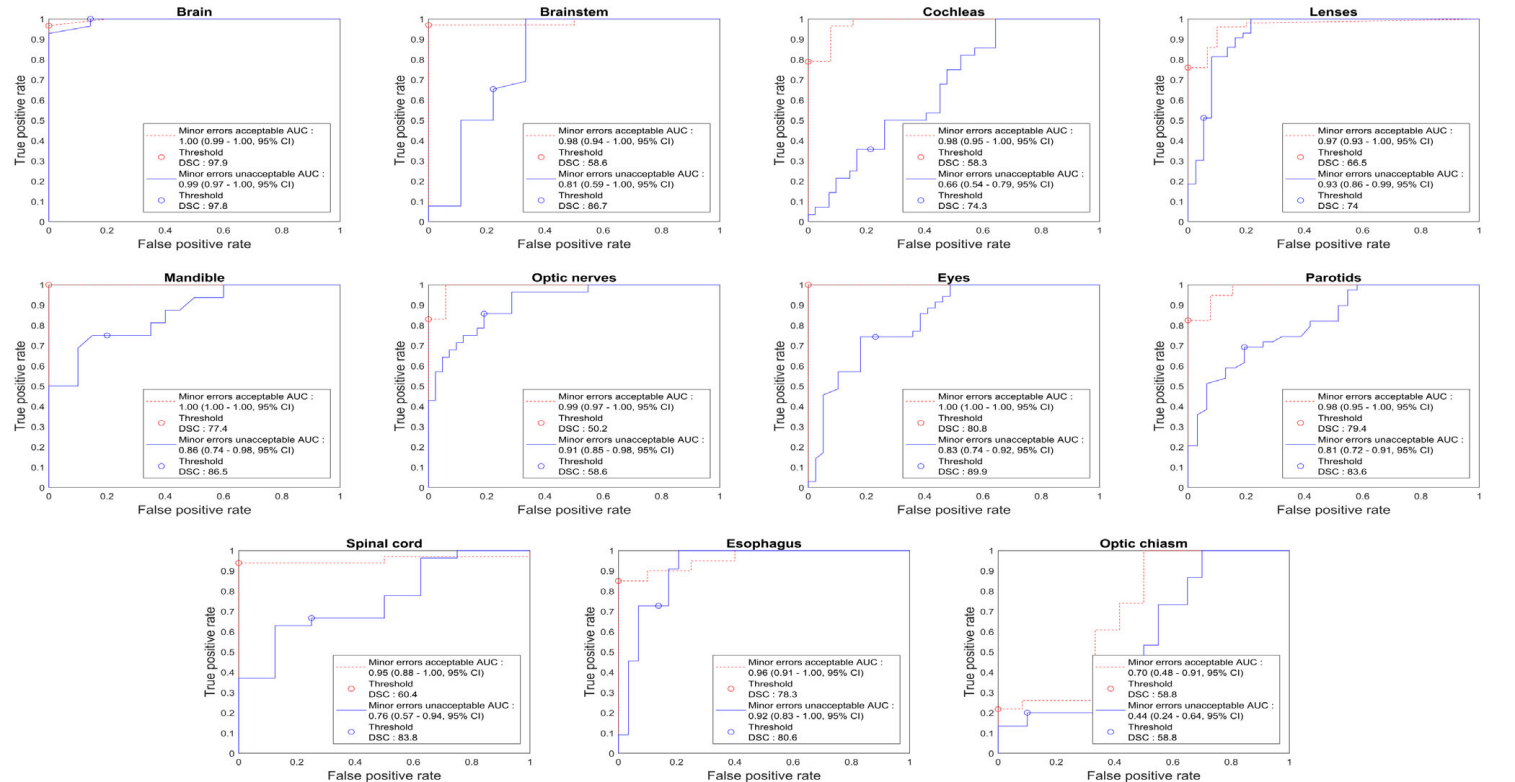
Receiver operating characteristic curves generated for each structure with 40 patients. 95% confidence interval (CI) for area under the curves were derived with the bootstrapping method. Dice similarity coefficients thresholds were derived and presented for both minor contouring errors acceptable and unacceptable scenarios. [Color figure can be viewed at http://wileyonlinelibrary.com]

The ROC curves based on DSC and physicians' scoring for the 40 patients are shown in Fig. 3. The average and minimum AUCs were 0.98 and 0.95 (excluding the optic chiasm) if minor errors were considered clinically acceptable. That is, for the scenario where we wish to only detect situations where major edits are needed. The average and minimum AUCs were 0.85 and 0.66 if minor errors were clinically unacceptable. The sensitivity and specificity based on the given DSC thresholds on the 20 patients were given in Table 7. If minor errors were clinically acceptable, the average sensitivity and specificity was 0.81 and 0.99, respectively, excluding the optic chiasm. If minor errors were considered clinically unacceptable, the sensitivity and specificity was 0.61 and 0.80, respectively, excluding the optic chiasm.

**Table 7:** The sensitivity and specificity of 20 patients for each structure with minor contouring errors acceptable and unacceptable scenarios with 95% confidence interval (CI) derived with the binomial test.

Structure	Minor contouring errors acceptable		Minor contouring errors not acceptable	
	Sensitivity (95% CI)	Specificity (95% CI)	Sensitivity (95% CI)	Specificity (95% CI)
Brain	0.80 (0.52–0.96)	1.00 (0.16–1.00)	0.92 (0.64–1.00)	0.75 (0.19–0.99)
Brainstem	0.94 (0.70–1.00)	1.00 (0.03–1.00)	0.77 (0.46–0.95)	0.75 (0.19–0.99)
Cochleas	0.72 (0.53–0.87)	1.00 (0.48–1.00)	0.08 (0.00–0.36)	0.95 (0.76–1.00)
Lenses	0.64 (0.44–0.81)	1.00 (0.74–1.00)	0.31 (0.14–0.52)	0.86 (0.57–0.98)
Mandible	0.92 (0.64–1.00)	1.00 (0.40–1.00)	0.75 (0.35–0.97)	0.78 (0.40–0.97)
Optic nerves	0.87 (0.66–0.97)	0.91 (0.59–1.00)	0.87 (0.60–0.98)	0.79 (0.54–0.94)
Eyes	1.00 (0.88–1.00)	1.00 (0.63–1.00)	0.53 (0.28–0.77)	0.74 (0.49–0.91)
Parotids	0.77 (0.58–0.90)	1.00 (0.40–1.00)	0.62 (0.38–0.82)	0.77 (0.46–0.95)
Spinal cord	1.00 (0.80–1.00)	NA*	0.71 (0.42–0.92)	0.67 (0.09–0.99)
Esophagus	0.47 (0.21–0.73)	1.00 (0.40–1.00)	0.50 (0.19–0.81)	0.89 (0.52–1.00)
Optic chiasm	0.07 (0.00–0.34)	1.00 (0.29–1.00)	0.17 (0.00–0.64)	1.00 (0.72–1.00)

NA*: No major contouring errors presented.

**Figure 4:**
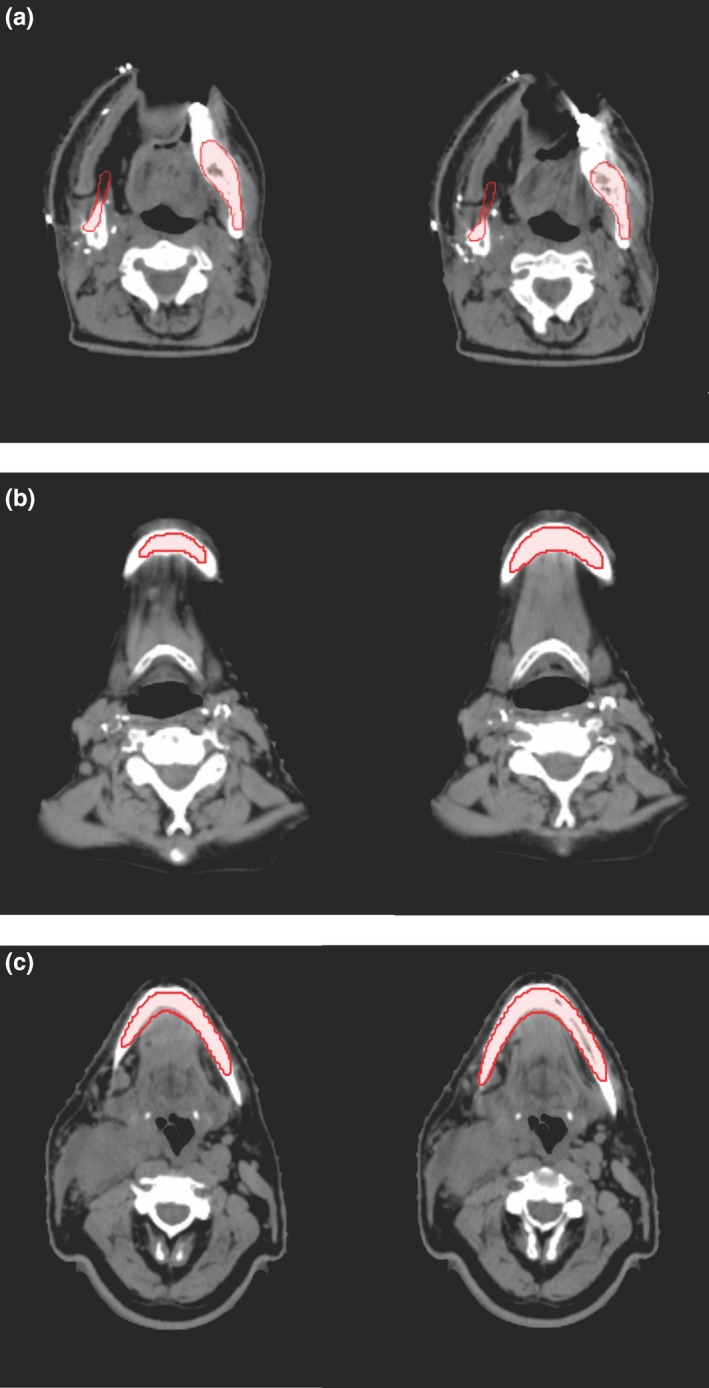
Multiatlas‐based autocontouring system mandible contours with errors were tested with the error detection system. Two consecutive slices around the most erroneous region were presented for each case, and the results were (a) major error detected, (b) minor error detected, and (c) minor error not detected. [Color figure can be viewed at http://wileyonlinelibrary.com]

The MACS mandible contours with errors are demonstrated in Fig. 4. Figure 4(a) shows the major error in the mandible which was detected by our system (DSC = 74.0). Figure 4(b) shows the minor error case detected by our system (DSC = 83.5), and Fig. 4(c) shows the minor error case undetected by our system (DSC = 86.7). As shown in Figs. 4(b) and 4(c), most of the minor error cases are only required to modify very small volumes which have a small impact on DSC, so the DSC distributions of no errors and minor errors are difficult to be distinguished.

## 4 Discussion

We have demonstrated that a CNN‐based architecture can accurately contour normal structures in the head and neck region. CNN architectures are preferred to have a fixed‐size input. However, the scan range and the number of slices significantly vary according to clinical protocols and patients' height. To address this problem, we have used a unique approach of using a CNN‐based classification architecture, when the DeepMind model was built to have a partial 3D CT scan with 21 slices as an input to contour a single‐CT slice. Our approach allows us to flexibly choose any 2D or 3D CNN‐based segmentation architectures, so we could choose the segmentation architectures based on the performance and/or the GPU memory availability. As we trained each organ contouring algorithm independently, we would be able to retrain any poorly performing architecture independently later with an advanced architecture or newly collected data. Furthermore, we can think a classification‐segmentation combination for a single organ as a module and apply it to contour the organ for other sites, such as the esophagus for thoracic patients. A disadvantage to this approach is that multiple models are used to generate contour predictions requiring additional time to predict all contours; however, our approach still manages to contour 16 structures on a CT scan of 160 slices in 2 min using a single GPU.

The accuracy of MACS contours was strongly associated with DSC between CNN‐based model contours and MACS contours. This association indicates that our CNN‐based model can effectively identify the major errors in the MACS contours. To date, most of the automated contouring error detection techniques were developed with machine learning algorithms using features or shapes of contouring structures^16^, ^17^, ^36^ and the relative positions^18^ of the contours. Chen et al.,^18^ showed that their geometric attribution‐based contouring error detection algorithms for the brain, brainstem, parotids, optic nerves, and optic chiasm contours can achieve the average sensitivity of 0.786–0.831 and the average specificity of 0.878–0.951. These show that our system has similar accuracy MACS contouring errors for the previously developed machine learning‐based algorithms. Furthermore, because most of the significant errors from MACS contours were specifically caused by irregular patient positions or abnormally large tumors, erroneous MACS contours in these cases still have reasonably good shapes and relative positions as shown in Fig. 4. Therefore, our CNN‐based contouring verification system has a strength in detecting significant errors in such cases over the other machine learning‐based error detection algorithms.

### 4.A. Model accuracy

The number of training, validation, and test data we used to train and evaluate the model is the largest among deep learning‐based head and neck autocontouring studies up‐to‐date.^26^ Although the accuracy of the internal dataset does not seem to be superior to other CNN‐based models, the end‐to‐end comparison shows that the accuracy of our model is almost equivalent to that from DeepMind, which has near expert dosimetrist level accuracy, except for cochleas. Additionally, our model achieved similar or lower standard deviations compared with the DeepMind model and had no completely failed cases (DSC = 0) on the TCIA data. The consistency and robustness of a model are important characteristics as a quality assurance tool, and our model has a strength in these aspects.

**Figure 5:**
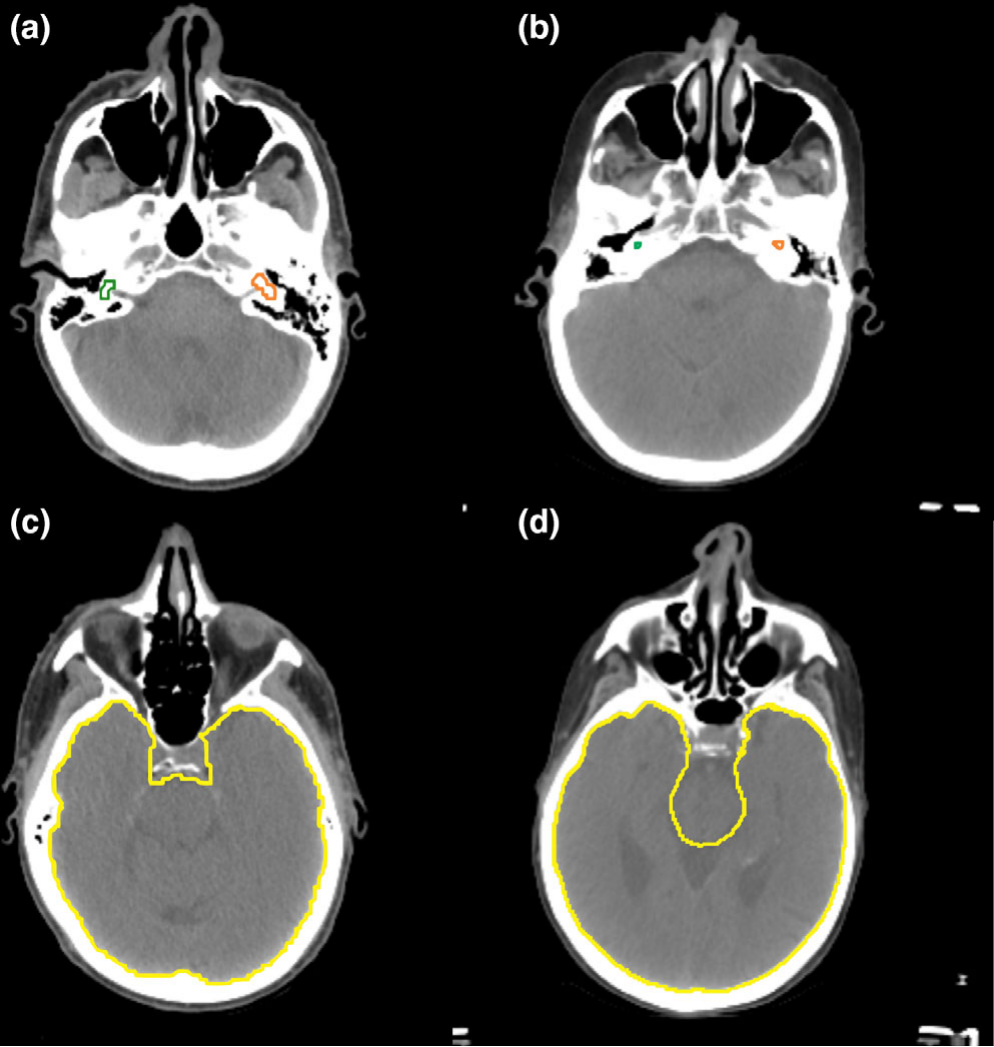
Physician‐drawn contours for cochleas (a), (b) and brain (c), (d). (a) and (c) were drawn by physicians at MD Anderson, and (b) and (d) were drawn by physicians working with DeepMind. [Color figure can be viewed at http://wileyonlinelibrary.com]

The accuracy of the cochlea contours from our CNN‐based model was significantly inferior to those from the DeepMind model. However, the volume of the cochlea was about three to four times larger for our model than that from the DeepMind model as shown in Figs. 5(a) and 4(b). The volume difference was due to differences in how clinicians contour the cochleas. At MD Anderson, the semicircular canals are included as part of the cochlea to reduce the risk of hearing loss from radiation treatment,^37^ while the DeepMind's contour does not include the semicircular canals. Therefore, most of the DeepMind physician's cochlea contours are completely covered by the cochlea contours from our model, and the true‐positive ratio, the volume ratio of the cochlea from the DeepMind model covered by the cochlea from our model, was 97.0% ± 5.0 (SD). Furthermore, the DSCs of the left and the right cochlea were 65.2% and 67.6%, respectively, with our physician‐drawn contours (Table 4), but were 39.5% and 42.2%, respectively, with the DeepMind physician‐drawn contours (Table 5). The accuracy of our model was still lower by about 10%, but considering the small volume of a cochlea, the difference would probably not significantly affect the final dose distribution of radiation treatment planning, especially if a small planning margin is created around the structure.

The definitions of other structures also differed somewhat between the two groups. The brain was defined to exclude the brainstem in the DeepMind contour, whereas the brainstem was a part of the brain in MD Anderson's contour, as shown in Figs. 5(c) and 5(d). These differences in contouring style underestimate the DSC of our CNN‐based model about 2% (Table 5) compared with the DSCs of the brain with our physician's contour (Table 4). This indicates that the actual differences in the accuracies of the brain would be less than 1 % between the two models.

### 4.B. Automatic verification of automatically generated contours

The specificity showed that major contouring errors can be confidently identified by measuring DSC between our CNN‐based contours and the target contours for most of the head and neck normal structures. Furthermore, the AUCs and the average sensitivity showed that the overall accuracy of these tool to identify major contouring errors is sufficient to be clinically implemented. Any major error in the optic chiasm, however, was difficult to be identified. For the optic chiasm, our CNN‐based model was neither consistent nor robust [mean DSC, 40.7% ± 13.9% (SD)]. The poor performance on contouring the optic chiasm was due to the very low contrast of the optic chiasm in CT images; even experts struggle to precisely draw the optic chiasm on CT, so MRI is recommended for contouring the optic chiasm.^38^ This difficulty can be seen in how the chiasm is drawn in clinical practice, with much variation in size and shape.

Although the AUCs and the sensitivity and specificity showed some potential to identify minor contouring errors for some structures, the relationship was not as strong as it was with major contouring errors. As we defined a minor contouring error to be a contour located in a right position but required a small shape modification, DSC, the geometric overlap over two contours, could not be a sensitive metric to measure the difference between well‐defined contours and contours requiring minor edits. Additionally, as minor errors were defined to be small variations in shapes of contours, implementing both our system and the feature‐based^16^ or the shape‐based contour verification tools^17^, ^36^ would improve the overall contouring verification accuracy.

One of the limitations of the automatic error verification study is that each contour was scored by only one radiation oncologist, so the study does not include the impact of interobserver variability. Because every radiation oncologist has slightly different ways to draw contours, it is possible that a contour scored to be “no error” can be scored as “minor error” by another radiation oncologist and vice versa. Similarly, some of the cases that we failed to predict the scores could have been successful if another radiation oncologist scores them, so further study with taking account of interobserver variability would be able to improve the overall robustness of our tool.

## 5 Conclusion

We have demonstrated that a CNN‐based autocontouring tool with near expert dosimetrist level accuracy for most of the head and neck normal structures can be developed using semiautomatically curated patient data. Furthermore, our model enables the detection of most of the major errors in the normal structures of the head and neck contours created by a clinically validated multiatlas‐based autocontouring tool.

## Conflict of interest

This work was partially funded by the National Cancer Institution and Varian.
